# Case report: Clinical characteristics and Genetical analysis of *HSD11B2* in three Chinese children with apparent mineralocorticoid excess: a case series

**DOI:** 10.3389/fendo.2024.1491825

**Published:** 2025-01-27

**Authors:** Yuan Ding, Ming Cheng, Bingyan Cao, Min Liu, Xuyun Hu, Di Wu

**Affiliations:** ^1^ Department of Endocrinology, Genetics, Metabolism, Beijing Children’s Hospital, Capital Medical University, National Centre for Children’s Health, Beijing, China; ^2^ Ministry of Education (MOE) Key Laboratory of Major Diseases in Children, Beijing Children’s Hospital, Capital Medical University, National Center for Children’s Health, Beijing, China; ^3^ Beijing Key Laboratory for Genetics of Birth Defects, Beijing Children’s Hospital, Capital Medical University, National Center for Children’s Health, Beijing, China

**Keywords:** apparent mineralocorticoid excess, hypokalemia, low-renin hypertension, renal calcification, case series

## Abstract

**Background:**

Apparent Mineralocorticoid Excess (AME) is a rare autosomal recessive disorder, characterized by a notably complex diagnostic process. To date, the majority of documented cases have been presented as individual case reports. This article aims to enhance the understanding of the course and prognosis of AME, by detailing the management protocols employed for patients with genetically confirmed diagnoses.

**Methods:**

An analysis comprising three cases and a review of relevant literature were conducted to synthesize the insights and experiences derived from gathering clinical and laboratory data on patients.

**Results:**

All three patients were born to non-consanguineous parents, were small for gestational age and exhibited severe hypokalemia, metabolic alkalosis, hypertension, nephrocalcinosis, and hypercalciuria. The glomerular filtration rate was normal in all cases. One patient experienced complications related to hypertension. Genetic analysis revealed biallelic recessive variations in the *HSD11B2* gene in all three patients. Treatment with oral spironolactone and potassium chloride resulted in the normalization of both blood pressure and serum potassium levels in all patients.

**Conclusion:**

This study presents the diagnostic and treatment experiences of three Chinese pediatric patients with AME type I. Through our analysis, four novel variants of the *HSD11B2* gene were identified, thereby enhancing the genotype-phenotype spectrum associated with AME. Early genetic testing in patients suspected of having AME is beneficial for facilitating prompt diagnosis and the implementation of standardized treatment protocols. Such measures are essential for the prevention or mitigation of target organ damage, as well as for the reduction of associated morbidity and mortality

## Introduction

Apparent mineralocorticoid excess (AME, #OMIM 614232), also known as cortisol 11β- hydroxysteroid deshydrogenase deficiency, is an autosomal recessive disorder caused by mutations in the 11β-hydroxysteroid dehydrogenase type 2 (*HSD11B2*) gene (1). This condition was first reported by Werder et al. in 1977 and can manifest in either neonates or adults, characterized by low birth weight, developmental delay, low-renin hypertension, hypokalemia, metabolic alkalosis, and increased risk of chronic kidney disease (2). Based on the degree of reduced HSD11B2 enzyme activity, AME is classified into type I and type II (3). This study retrospectively reports on the diagnosis and treatment of three children who presented with hypertension, hypokalemia, and renal calcifications early in life but had normal renal function, aiming to deepen the understanding of the natural course and prognosis of apparent mineralocorticoid excess, and to summarize experiences and insights through a literature review.

## Methods

We retrospectively collected clinical, laboratory, imaging, and molecular data from three patients diagnosed with AME, who all came from non-consanguineous parents. This study was approved by the Ethics Committee of Beijing Children’s Hospital. Parents of each patient were informed about the study procedure and provided informed consent prior to the collection of peripheral blood samples for whole-exome sequencing (WES). All detected variants were verified using Sanger DNA sequencing. Variants were annotated using the ANNOVAR software (http://annovar.openbioinformatics.org/en/latest/) and cross-referenced with multiple databases including gnomAD, 1000 Genome, ESP6500, dbSNP, ExAC, ClinVar, and HGMD. The criteria for selecting potentially pathogenic variants in the downstream analysis were as follows: 1) Variant read depth of more than 5, with a variant allele frequency of no less than 30%; 2) Exclusion if the variant frequency was more than 5% in the gnomAD, ExAC, 1000 Genomes, and ESP6500 databases; 3) Exclusion of synonymous variants if they were not found in the HGMD database. The pathogenicity of genetic variants was assessed using the American College of Medical Genetics standards, and predictions were made through computational methods and tools such as REVEL, SIFT, PolyPhen-2, MutationTaster, and GERP++.

## Case reports

### Case 1

A 12-year-old female was admitted to our hospital due to hypokalemia and hypertension. She was born term at 38 weeks, with a birth weight of 1500g (less than 10^th^ percentile). At 6 months of age, she was admitted to a local hospital for fever and vomiting, during which hypokalemia and elevated blood pressure were observed (specific values not provided). Consequently, she began oral potassium chloride therapy. Over time, she experienced growth and developmental delays, and hypertension. Previously, the patient was diagnosed with epilepsy at an outside hospital after experiencing one seizure (specific details of diagnosis and treatment are unspecified). No antiepileptic drugs were given, and there have been no subsequent seizures. The lowest recorded potassium level was 1.23 mmol/L. Despite administration of oral potassium chloride in combination with captopril, blood pressure control remained suboptimal, with systolic blood pressure ranging from 140 to 170 mmHg and diastolic blood pressure between 100 and 110 mmHg.

Her height and weight lagged substantially behind in those of same-age female peers. Echocardiography indicated left ventricular hypertrophy, with left ventricular systolic function at the lower end of the normal range, left ventricular ejection fraction between 50-60%, widened aortic sinuses, and minimal aortic valve regurgitation. Head MRI indicated localized cerebral atrophy and cystic changes in the left parietal lobe, as well as keratin hyperplasia. Additionally, mild renal injury was found, along with hypercalciuria and nephrocalcinosis. She began oral spironolactone to maintain serum potassium level, alongside metoprolol therapy. At the last follow-up, the patient was 14 years and 10 months old. Despite the dosage of spironolactone being increased to 6.8 mg/kg/day, serum potassium levels could not be stabilized within normal range. Moreover, blood pressure control remained poor, with systolic blood pressure ranging from 135 to 140 mmHg and diastolic blood pressure between 80 and 90 mmHg. Following the adjustment from spironolactone to eplerenone (gradually increased to 5 mg/kg/day), blood pressure control improved, with systolic blood pressure fluctuations between 120 and 125 mmHg and diastolic blood pressure fluctuations between 70 and 80 mmHg. Nevertheless, there was no improvement in the left ventricular hypertrophy, the left ventricular ejection fraction remained normal, yet there was a trend indicating worsening renal injury. The specific values of physical examination and laboratory findings are detailed in **Table 1**.

**Table 1:** Clinical characteristics and main auxiliary examination results of the three patients during consultation and follow-up.

	Case 1	Case 2	Case 3
Gender	female	female	male
Age at diagnosis (year)	12	8.9	3.3
Gestational age (week)	38	39	40
Birth weight (g)	1550	2100	2500
First visit			
Height (cm, SDS)	124.1, -4.38	116.4, -3.05	86.2, -3.87
Weight (kg, SDS)	17.5, -4.69	21.5, -1.65	10, -3.52
BMI (kg/m^2^, SDS)	11.36, -3.94	15.87, 0.18	13.46, -2.04
Systolic blood pressure (mmHg)^a^	155	142	121
Diastolic blood pressure (mmHg)^a^	110	91	83
Potassium (mmol/L)	3.14	2.28	3.18
HCO_3_^-^ (mmol/L)	27.2	31.3	26.6
Creatinine (mg/dl)	51.6	32.3	29.1
eGFR^b^ (ml/min/1.73m^2^)	87.87	131.54	108.12
Renin (pg/ml/h)	3.23	1.50	1.00
Aldosterone ^c^(ng/dl)	7.05	5.62	6.33
Urine calcium/creatinine (mg/mg)	0.44	1.11	0.63
Urine protein/creatinine (mg/mg)	0.63	0.3	0.1
Nephrocalcinosis	+	+	+
Follow-up			
Age at last visit (year)	14.8	9.1	3.6
Height (cm, SDS)	142, -3.19	118.1, -2.87	92, -2.4
Weight (kg, SDS)	26.4, -4.61	22.5, -1.46	10.8, -3.49
BMI (kg/m^2^, SDS)	13.1, -3.46	16.1, 0.28	12.8, -2.79
blood pressure (mmHg)	120/80	108/70	100/65
Normal blood pressure(mmHg)^d^	116/74	108/68	99/62
Left Ventricular Hypertrophy	+	–	–
Hypertensive retinopathy	–	–	–
Potassium (mmol/L)	3.52	3.9	3.51
Creatinine (mg/dl)	68	42	24.4
eGFR (ml/min/1.73m^2^)	76.22	102.63	137.6
Urine calcium/creatinine (mg/mg)	0.078	0.22	0.85
Treatment			
Spironolactone (mg/kg/day)	5 (Eplerenone)	5.5	5.5

GRF, glomerular filtration rate; SDS, standard deviation score

a Hypertension was defined based on the latest pediatric (age-, sex-, and height-specific percentile-based) hypertension guidelines: three non-consecutive day measurements of systolic and/or diastolic blood pressure ≥ the 95th percentile for the same age, sex, and height.

b eGFR = K × height (cm)/creatinine (µmol/L), where K = 36.5 for ages 1-12.

c Aldosterone(clinostatism) reference range:1-16ng/dl

d 90th percentile blood pressure for age, sex, and height.

### Case 2

An 8-year-old girl was admitted to our hospital due to “Bartter syndrome”. She was born term at 39 weeks, with a birth weight of 2100g (less than 10^th^ percentile). She was admitted to a regional hospital for the first time at the age of 22 months because of polydipsia and polyuria. She was suspected to have Bartter syndrome due to hypokalemia and metabolic alkalosis, but no further evaluation was done. She was continuously treated with oral indomethacin and potassium chloride until 8 years and 11 months of age, with potassium levels ranging from 2.1 to 3.3 mmol/L, accompanied by growth retardation. Her height and weight also lagged significantly behind in those of same-age female peers. Hypertension was detected through blood pressure monitoring (range of blood pressure 135-150/80-100 mmHg), and she also exhibited hypercalciuria and nephrocalcinosis. After whole exome gene sequencing, she was diagnosed with AME. Indomethacin was temporarily discontinued, and oral spironolactone was introduced. This intervention restored normal potassium levels, allowing for the gradual reduction and eventual cessation of oral potassium chloride.

When she was 9 years and 1 month old, her height was 118.1 cm, weight was 22.5 kg, and her blood pressure was controlled at approximately 120/80 mmHg. She was prescribed spironolactone at a dosage of 5.5 mg/kg/day, which maintained her serum potassium levels within normal limits. Furthermore, the urine calcium to creatinine ratio has decreased from previous assessments, suggesting a reduction in urinary calcium excretion. **Table 1** present the specific values of physical examination and laboratory findings.

### Case 3

A 3-year-old boy presented to our hospital due to growth retardation since early childhood. He was born term at 40 weeks, with a birth weight of 2500g (less than 10^th^ percentile). At 16 months of age, he was admitted to a local clinic for vomiting and diarrhea. He was observed to have hypokalemia, metabolic alkalosis and nephrocalcinosis, but no further evaluation was done. He began oral potassium chloride, but his serum potassium levels could only be maintained between 2.1 and 2.7 mmol/L. Following the consultation, the persistent elevation of systolic blood pressure led us to suspect an adrenal disorder, and whole exome sequencing subsequently validated the diagnosis of AME.

The patient, currently aged 3 years and 7 months, has a height of 92 cm and a weight of 10.8 kg. He is now administered oral spironolactone at a dosage of 5.5 mg/kg/day, which has effectively controlled the blood pressure within the range of 95-100/60-65 mmHg while maintaining normal serum potassium levels. Moreover, the urinary calcium to creatinine ratio has also decreased compared to previous measurements. **Table 1** describe the clinical data of the patient at the time of initial presentation and during follow-up, respectively.

### Clinical characteristics and molecular findings

All three patients were born small for gestational age and exhibited postnatal growth retardation. However, their intellectual and motor development was normal. Two patients presented with vomiting, diarrhea, polydipsia, and polyuria, while another was discovered to have hypokalemia and metabolic alkalosis during a visit for fever. All patients presented with hypertension but did not exhibit symptoms such as headache or dizziness. Hypercalciuria and nephrocalcinosis were common findings. All three patients showed no abnormalities in routine blood, urine, and stool tests, with normal liver and thyroid function, glycated hemoglobin, and 24-hour urinary electrolytes (sodium, potassium, chloride, phosphorus). Funduscopic examinations for all three patients did not reveal any signs of hypertensive retinopathy. Furthermore, oral potassium chloride alone was insufficient for all patients to stabilize their serum potassium levels within normal limits.

Case 1 was homozygote, and Case 2 and Case 3 were compound heterozygotes of *HSD11B2* variants, confirmed by parental sanger DNA sequencing (seen in **Table 2**). The p.V217A variant, although rated as “uncertain” by ACMG, is predicted to be 0.753, damaging, benign, disease causing, and 5.15 according to REVEL-score, SIFT, PolyPhen-2, MutationTaster, and GERP++.

**Table 2:** The molecular information of three patients with AME.

Case	Nucleotide	Amino acid	Exon	Status	Origin	ACMG
1	c.650T>C	p.V217A	3	homozygous	biparental	uncertain
2	c.763dup	p.V255Gfs*102	4	compound heterozygous	maternal	likely pathogenic
	c.204_226del	p.L69Afs*15	1		paternal	likely pathogenic
3	c.662C>T	p.A221V	3	compound heterozygous	maternal	likely pathogenic
	c.1017C>A	p.Y339*	5		paternal	likely pathogenic

## Discussion

AME is a rare monogenic hereditary disorder caused by variants in the *HSD11B2* gene. To date, fewer than 100 cases have been reported worldwide, and the prevalence of the condition remains uncertain. Patients are often offspring of consanguineous families with homozygotes or are more commonly found in certain ethnic populations, such as Native Americans and the Omani population (4, 5). Type I AME is characterized by a complete or severely reduced activity of 11β-HSD2, resulting in severe clinical symptoms that manifest in childhood, poor prognosis, and a high mortality rate. This type often occurs in offspring of parents who are related by blood, and the mutations are typically homozygous. Type II AME presents with a milder clinical phenotype, with onset in adulthood and only mild laboratory abnormalities, sometimes even without electrolyte disturbances, resembling primary hypertension. The roles of the mineralocorticoid receptor and the HSD11B2 enzyme in primary hypertension are increasingly being recognized (6). The three patients in this study presented with an early age of onset, severe low birth weight, poor growth, no catch-up growth, and emaciation, but with normal intelligence. They exhibited classic features of metabolic alkalosis, low renin, low aldosterone, hypokalemia, and hypertension. The mean arterial pressure of all subjects was higher than the 90th percentile for their age, gender, and height. Consistent with previous literature, Case 1 exhibited not only the typical manifestations of increased mineralocorticoid activity but also secondary renal dysfunction, left ventricular hypertrophy, and widespread atherosclerosis of major arteries, leading to severe organ damage. Upon admission, treatment with spironolactone for blood pressure reduction and potassium-sparing therapy resulted in a significant improvement in hypertension. The blood pressure measurements of all patients approached normal levels, and the incidence of hypertension-related complications was low. Additionally, the use of spironolactone combined with low-dose potassium supplementation alleviated hypokalemia and polyuria symptoms, and improved growth in the patients.

11β-HSD2 is a key enzyme in the metabolism of cortisol. Under normal circumstances, after aldosterone binds to the mineralocorticoid receptor (MR), the resulting ligand-receptor complex translocates to the cell nucleus where it interacts with hormone response elements (HREs), enhancing the transcription of genes that encode specific aldosterone-induced proteins, thereby exerting effects such as sodium retention and potassium excretion. *In vitro* studies have shown that although cortisol and aldosterone have the same affinity for the MR, the levels of cortisol in the body are 100 to 1000 times higher than those of aldosterone (7). However, aldosterone is the physiological agonist for the MR, capable of converting biologically active cortisol into inactive cortisone, thereby protecting the MR from activation by cortisol (1). When mutations in the HSD11B2 gene lead to the absence or reduced activity of 11β-HSD2, the levels of NADH decrease, and the cortisol-MR complex becomes activated for transcription, mimicking the actions of aldosterone (8). The loss of catalytic activity and affinity for substrates and cofactors is considered the fundamental mechanism leading to the loss of enzyme activity and the occurrence of AME (9). The absence or reduced activity of 11β-HSD2 prevents the conversion of cortisol to cortisone, resulting in an increased ratio of cortisol to cortisone in the blood and urine. The level of this ratio can be used to assess the extent of the deficiency in 11β-HSD2 enzyme activity (10). Patients with AME often experience mild to moderate intrauterine growth retardation and are born with low body weight. This is because 11β-HSD2 plays a role in the development of the placental barrier. Its primary function is to limit fetal exposure to maternal cortisol, protecting the fetus from the effects of the physiological increase in maternal glucocorticoids during pregnancy. Mutations in the *HSD11B2* gene lead to a lack of 11β-HSD2 in the placenta, allowing excessive maternal glucocorticoids to cross the placenta and inhibit fetal growth (11). The polyuria and polydipsia observed in AME are believed to be caused by defects in urine concentration (partial nephrogenic diabetes insipidus), which are associated with persistent hypokalemia, or due to chronic overactivation of the MR by cortisol, leading to reduced sensitivity of the renal tubules to antidiuretic hormone. The mechanisms underlying nephrocalcinosis are not well understood. In this case, typical causes of nephrocalcinosis such as hypercalcemia, hypercalciuria, and renal tubular acidosis have been ruled out. We propose that the renal calcification observed in this patient and others might be associated with dystrophic calcification in areas of interstitial fibrosis. The development of nephrocalcinosis and renal cysts may be related to long-term hypokalemia, resulting in dystrophic calcification in areas of interstitial fibrosis (7). We speculate that the renal calcification observed in three patients may be related to dystrophic calcification in areas of interstitial fibrosis. The three patients in this paper were all born with low birth weight and have had severe developmental delay since birth. All three patients have nephrocalcinosis, with two patients exhibiting polyuria and polydipsia. The urinary calcium-to-creatinine ratios were elevated in all three patients, with one patient eventually resolving this during follow-up. Currently, all patients have heights and BMIs below -2SD, consistent with the descriptions in the literature. Therefore, unlike renal calcification directly caused by other tubular diseases such as Bartter syndrome, Dent’s disease, and distal renal tubular acidosis, the mechanism underlying renal tubular calcification in AME may be more complex and cannot be solely attributed to hypercalciuria.

**Figure 1:**
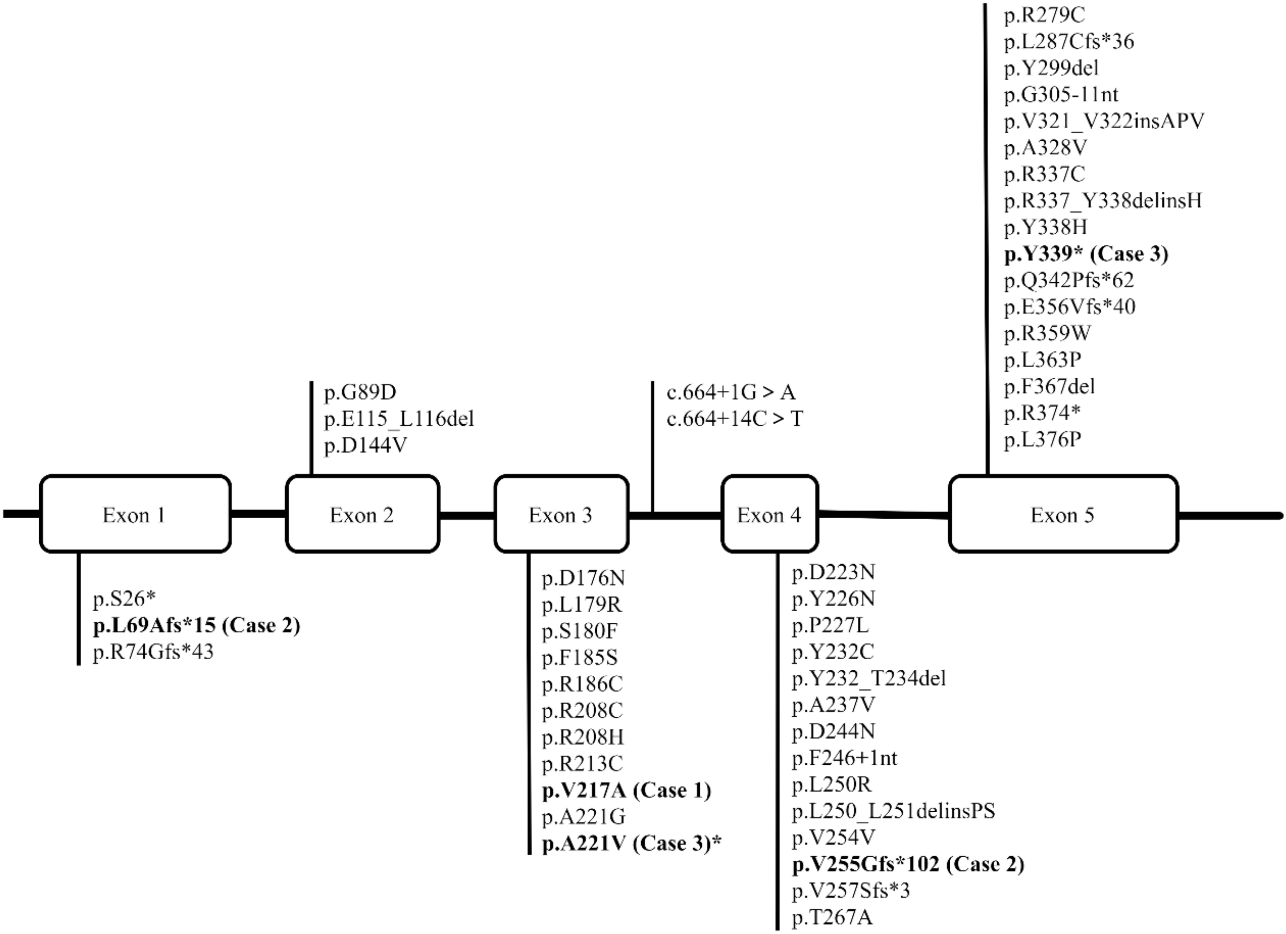
The figure depicts the structure of the human *HSD11B2* gene, which contains five exons. The reported variations are presented in regular font, while the variations we have identified in three patients are highlighted in bold type.

To date, over 40 pathogenic variants of the *HSD11B2* gene have been identified in patients with AME, with the majority being missense mutations concentrated in exons 3, 4, and 5. Other genetic abnormalities, including nonsense, splicing, insertion, and deletion variants, have also been reported, albeit at a lower frequency (http://www.hgmd.cf.ac.uk/docs/login.html) (summarized in **Figure 1**). In this study, all three patients exhibited reduced levels of plasma aldosterone, consistent with the diagnosis of AME.

Previous studies have elucidated the close relationship between the genotype, structure, and phenotype in AME (12). The severity of the AME phenotype, whether severe or mild, is primarily determined by the extent of loss or residual activity of 11β-HSD2 caused by *HSD11B2* variants. Patients who are homozygous for *HSD11B2* mutations that completely or nearly completely abolish 11β-HSD2 activity typically present with severe AME phenotypes in early childhood. In contrast, individuals who are heterozygous for *HSD11B2* mutations resulting in partial loss of 11β-HSD2 activity may exhibit a milder AME phenotype, which can manifest during adolescence or early adulthood (13). Literature indicates that homozygous *HSD11B2* mutations are significantly associated with low birth weight (14). In cases of compound heterozygosity, the clinical phenotype is the result of the combined effects of the two mutations. Relatives of probands carrying a single heterozygous mutation may only present with mild to moderate hypertension in adulthood (15). In recent years, an increasing number of clinical and biochemical evidences support the existence of mild AME type II in the general population, leading to some cases of hypertension being misclassified as idiopathic (16). In the present study, Case 1 is a patient with homozygous HSD11B2 mutations, low birth weight, and severe AME phenotype in early childhood, consistent with the literature description. Cases 2 and 3 both have compound heterozygous HSD11B2 mutations, with early onset and also present with low birth weight. However, only Case 1 exhibits severe hypertension, developing hypertensive heart disease, thickening of the intima-media of the abdominal aorta and bilateral carotid arteries with multiple plaques. Therefore, all three patients in this study are classified as AME type I.

The clinical manifestations of AME patients are complex and lack specificity. Without the ratio of blood/urinary cortisol to cortisone, clinical diagnosis can be relatively challenging. Instead, it relies on enzyme activity measurement or gene mutation analysis for definitive diagnosis (2). Differential diagnoses include Liddle syndrome, ectopic ACTH syndrome, exogenous drug and food intake (licorice and its derivatives, excessive consumption of grapefruit, etc., can all affect the activity of the 11β-HSD2 enzyme) (1). The therapeutic goal of AME is to lower blood pressure and correct hypokalemia. The main treatment options include spironolactone and potassium supplementation. Spironolactone is an aldosterone receptor antagonist that competitively binds to the receptor, protecting it from the effects of excessive aldosterone. Early use of MR antagonists such as spironolactone, at doses of 2-12.5mg/kg/day, can normalize blood pressure, improve growth, reverse hypertensive retinopathy and left ventricular hypertrophy, and there is literature indicating that it can also reverse bilateral nephrocalcinosis (4, 16). Dexamethasone has been successfully used to lower blood pressure in adult AME patients, with an initial dose of 1.5-2mg/day, followed by a maintenance dose of 0.5mg/day. However, dexamethasone alone is not always effective in controlling hypertension (17, 18).

Although 11β-HSD2 is also present in the colon, the kidney is the primary site of 11β-HSD2 enzyme activity. Therefore, there have been reports of kidney transplantation: in two adult patients with AME and renal failure, the clinical symptoms of AME disappeared after kidney transplantation, spironolactone treatment was discontinued, and both low renin hypertension and hypokalemic alkalosis were alleviated (19). It is worth mentioning that during organ transplantation, usually immunosuppressive drugs are required. The most used ones are calcineurin inhibitors (CNI) which interestingly provoke Gordon-like syndrome, increasing blood potassium (20). Due to chronic hypertension being associated with significant complications and mortality, early kidney transplantation can reduce end-organ damage and prolong the lifespan of AME patients. In this study, although patient 1 had been previously treated with spironolactone and oral potassium chloride, irregular medication use resulted in difficult blood pressure control. After diagnosis, the three patients strictly followed the treatment plan, adjusted on time, and are currently only treated with oral spironolactone. Their blood potassium has returned to normal, and they have all discontinued the use of oral potassium chloride solution. Their blood pressure is now controlled within the normal range. This suggests that regular and proper medication use can achieve normal blood pressure control.

In conclusion, AME is a rare form of primary hypertension with relatively difficult clinical diagnosis. For patients with severe hypertension, hypokalemia, low renin, low aldosterone, and multi-organ damage, a step-by-step differential diagnosis should be conducted through biochemical tests and imaging studies. Although the analysis of 11β-hydroxysteroid dehydrogenase type 2 activity in peripheral blood leukocytes or skin fibroblasts is a key technique for definitive diagnosis of the disease, currently feasible genetic analysis aids in diagnosis, genetic counseling, and prenatal diagnosis. Our research presents three instances of Type I AME in children in our country, and found five novel mutation locations in the HSD11B2 gene, expanding the gene and phenotype spectrum of AME. Owing to the rarity of AME, it is necessary to pursue further follow-up studies on enduring complications, risk elements, and refinement of therapy.

## Data Availability

The raw data supporting the conclusions of this article will be made available by the authors, without undue reservation.
