# Supplementary material for: Case report: Clinical characteristics and Genetical analysis of HSD11B2 in three Chinese children with apparent mineralocorticoid excess: a case series
Source: Front Endocrinol (Lausanne). 2025 Jan 27;15:1491825. doi: 10.3389/fendo.2024.1491825 (PMC11807828; doi:10.3389/fendo.2024.1491825)

Supplementary figure 1. Partial genomic DNA sequences from the *HSD11B2* genes of three patients and their parents.

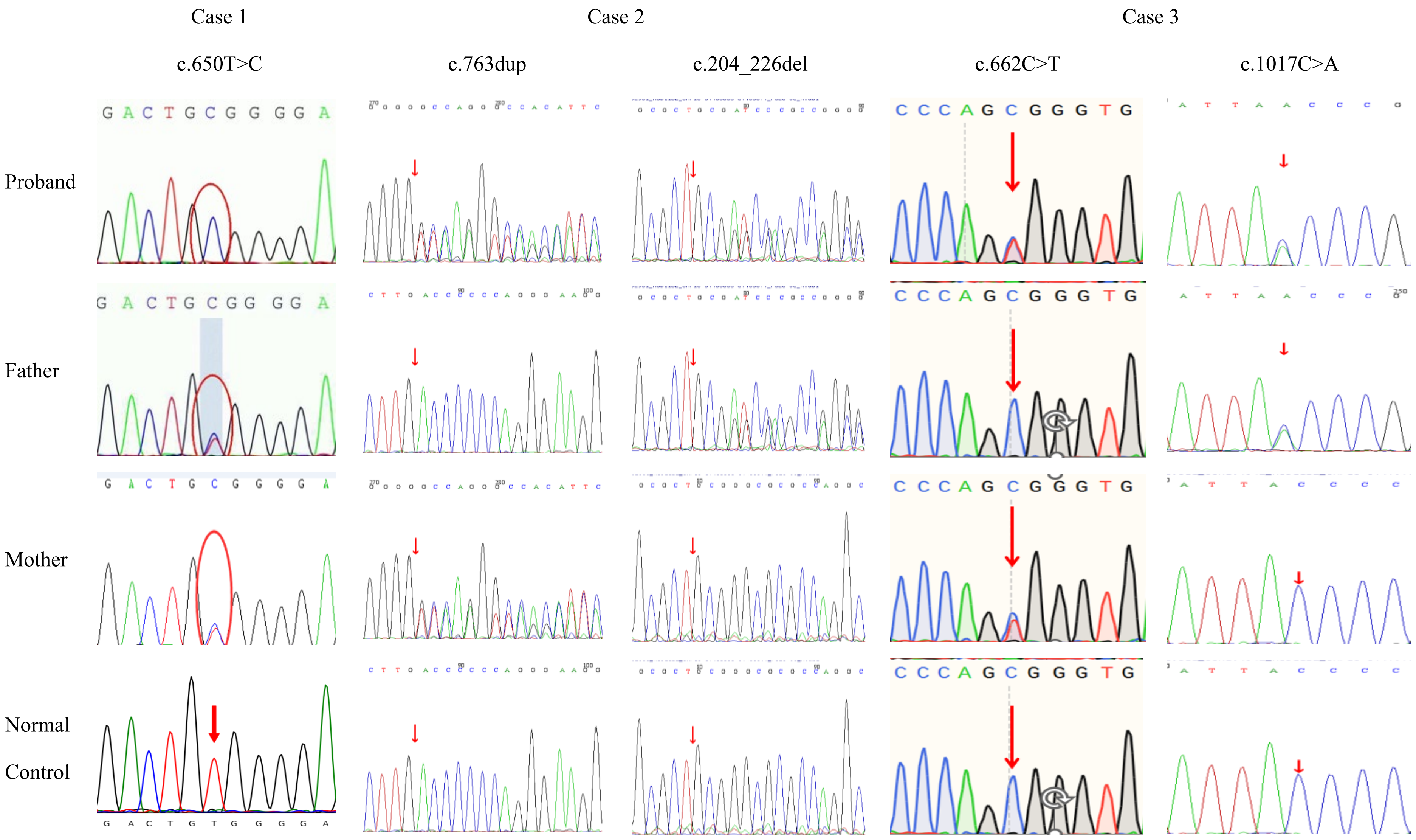

Supplement: Supplementary file 1 [file Image1.pdf]
